# Osteocalcin Serum Levels in Gestational Diabetes Mellitus and Their Intrinsic and Extrinsic Determinants: Systematic Review and Meta-Analysis

**DOI:** 10.1155/2018/4986735

**Published:** 2018-12-30

**Authors:** Raigam J. Martinez-Portilla, Jose R. Villafan-Bernal, Diana L. Lip-Sosa, Eva Meler, Jordi Clotet, Francisco J. Serna-Vela, Sergio Velazquez-Garcia, Leopoldo C. Serrano-Diaz, Francesc Figueras

**Affiliations:** ^1^Fetal i+D Fetal Medicine Research Center, BCNatal-Barcelona Center for Maternal-Fetal and Neonatal Medicine (Hospital Clínic and Hospital Sant Joan de Déu), IDIBAPS, University of Barcelona, Spain; ^2^Maternal-Fetal Medicine and Therapy Research Center Mexico in behalf of the Iberoamerican Research Network in Translational, Molecular and Maternal-Fetal Medicine, Mexico; ^3^Mexican Consortium of Biomedicine, Biotechnology and Health Dissemination-Consortium BIO2-DIS, Mexico; ^4^CONACYT Researcher at the Department of Surgery, Health Science Center, Autonomous University of Aguascalientes, Mexico; ^5^Center for Health Sciences, Autonomous University of Aguascalientes, Mexico; ^6^CIMeTA Research Unit-ISSEA, Mexico; ^7^Women's Hospital of the State of Aguascalientes-ISSEA, Mexico; ^8^Center for Biomedical Research on Rare Diseases (CIBER-ER), Madrid, Spain

## Abstract

**Background:**

Undercarboxylated osteocalcin (ucOC) increases insulin release and insulin resistance in mice. In humans, evidence is scarce but a correlation of ucOC and total osteocalcin (tOC) with glycemic status markers has been demonstrated. The relationship of ucOC and tOC with gestational diabetes mellitus (GDM) has been even less characterized.

**Objective:**

To assess the mean difference of tOC and ucOC serum concentrations among nondiabetic pregnant women and women diagnosed as GDM in the second trimester of pregnancy and to determine the possible intrinsic and extrinsic contributors to this difference.

**Methods:**

A systematic search was performed to identify relevant studies published in English and Spanish using PubMed, SCOPUS, ISI Web of Knowledge, and PROSPERO database for meta-analysis. Observational studies measuring mean serum levels of osteocalcin among GDM, with at least 10 subjects analyzed in each group were selected. Mean difference (MD) by random effects model was used. Heterogeneity between studies was assessed using Cochran's Q, H, and *I* ^2^ statistics.

**Results:**

From 38 selected studies, 5 were retained for analysis for a total of 1119 pregnant women. Serum concentrations of tOC were not significantly different among women with GDM and nondiabetic pregnant controls (MD: 1.56; 95% CI: −0.70 to 3.82; *p* = 0.175). Meanwhile, ucOC serum levels were significantly higher among women with GDM (MD: 1.17; 95% CI: 0.24 to 2.11; *p* = 0.013). The only factor influencing tOC was the UV index, showing a reduction in mean difference between GDM and controls when exposed to higher concentrations of UV rays.

**Conclusions:**

This meta-analysis provides evidence to support the use of ucOC as a potential marker for GDM rather than tOC, yielding very little variability among studies and no difference among methods or brands used for its analysis.

## 1. Introduction

Gestational diabetes mellitus (GDM) is defined as any glucose impairment with first onset or recognition during pregnancy [1]. With a prevalence within 2 to 6%, which is increasing worldwide, this condition represents a leading cause of adverse perinatal outcomes [2], with delayed effects both for women and children [3, 4]. Diagnosis of GDM usually happens in an advanced gestational age, limiting preventive strategies. Development of prediction models for GDM is a challenge to contemporary fetal-maternal medicine.

Osteocalcin (OC) is a bone-derived protein that takes part in bone metabolism [5]. About 10% to 30% of osteocalcin is released into the main circulation, which is later cleared by the liver and kidney [6–8]. The three primary forms of OC are carboxylated (cOC), undercarboxylated (ucOC), and total osteocalcin (tOC) [9]; these three biochemical markers can be separately measured in blood by different methods such as RIA (radioimmunoassay), IRMA (immunoradiometric assay), ELISA (enzyme-linked immunoassay), or electrochemiluminescence immunoassay (ECLIA).

From a clinical point of view, tOC and ucOC serum levels are lower among patients with type 1 and type 2 diabetes [10–27], while only a few studies have assessed the mean concentration of cOC suggesting increased levels in individuals with type 2 diabetes [26]. Some studies have shown that ucOC increases insulin secretion and proliferation of pancreatic beta-cells. It also increases adiponectin secretion from adipose tissue, reducing fat mass and increasing energy expenditure by increasing the expression of genes involved in beta-oxidation [28]. Nonetheless, gestational diabetes mellitus (GDM) is a different entity defined as glucose intolerance resulting in hyperglycemia of variable severity with onset or first recognition during pregnancy [1]. Maternal physiological changes during pregnancy lead to a decrease in fasting glucose at early pregnancy and continuously during gestation, together with a marked decline in insulin sensitivity during second and third trimester [29, 30]. One study performed in pregnant women demonstrated that tOC serum levels are higher during the first trimester of pregnancy in women subsequently developing GDM when compared to controls [31]. However, studies measuring tOC and ucOC during second trimester in nonprevious diabetic women have yielded controversial results [32, 33].

Defining the changes in serum levels of proteins or hormones related to insulin resistance or glucose tolerance such as tOC and ucOC among women developing GDM is vital for adequate future prediction models aimed to determine which women are at risk for developing this disease [34]. Indeed, a model combining maternal characteristics plus insulin-resistance-related proteins could lead to effective predictive algorithms for GDM [35].

The aim of this systematic review and meta-analysis is to quantify the changes of tOC and ucOC serum concentrations in women diagnosed with GDM in the second trimester of pregnancy and to determine the possible intrinsic and extrinsic determinants of these changes.

## 2. Methods

### 2.1. Protocol Registration

Before data extraction, the project was registered in the PROSPERO international prospective register of systematic reviews (registration number: CRD42018091727).

### 2.2. Eligibility Criteria, Information Sources, and Search Strategy

A systematic search was performed using PubMed, EBSCO, ISI Web of Science, and PROSPERO database for meta-analysis to identify relevant studies published in English or Spanish, without publication time restrictions. Institutional review board approval is not required for systematic reviews in our institution. References of relevant publications were manually searched for additional potentially relevant studies. The first search was run on May 20, 2017. Afterward, an update was extended until March 23, 2018.

This review was carried out adhering to the Meta-analysis of Observational Studies in Epidemiology (MOOSE) guidelines [36] and the Preferred Reporting Items for Systematic Reviews and Meta-Analyses (PRISMA) guidelines for systematic reviews and meta-analysis [37, 38]. Two independent evaluators (R.M. and R.V.) assessed abstracts identified as relevant, both blinded to authorship, authors' institutional affiliation, and study results. If the abstract fulfilled inclusion criteria, full-text articles were then reviewed. A third investigator (F.F.) independently settled any disagreement between evaluators. In case of relevant studies with missing information, corresponding authors were reached by e-mail to request such data.  Annex 1 of supplemental material details the search strategy and query syntaxes.

### 2.3. Study Selection

The inclusion criteria for this systematic review were observational studies (prospective or retrospective) on women diagnosed with GDM between 24 and 28 weeks of gestation and their respective nondiabetic pregnant controls, with at least ten women in each group and reporting serum levels of OC.

### 2.4. Data Extraction

The following information was extracted on a datasheet based on Cochrane Consumers and Communication Review Group's data extraction template [39]: author, year of publication, country where the study was conducted, study period, type of study, original inclusion and exclusion criteria, total number of patients included in the study, number of participants with GDM, number of nondiabetic pregnant women, osteocalcin quantification method, form of osteocalcin determined, mean maternal age at analysis, mean maternal body mass index (BMI), mean gestational age at measurement of OC, method of quantification, brand of the kit used for the measurement, and mean UV index as a surrogate of sun exposure.

### 2.5. Assessment of Risk of Bias

Two reviewers (R.M. and D.L.) independently assessed the quality of the selected studies. Quality assessment of observational studies was carried out using the Newcastle-Ottawa scale for case-control studies and cohort studies. Each study was judged on three dimensions: the selection of the study groups, the comparability of the groups, and the ascertainment of the exposure. One star was given for each signaling question among each dimension. For a total of 9 possible stars, studies with 7 or more stars were considered as high quality [40].

### 2.6. Data Analysis

Extracted results were pooled in the meta-analysis. Data analysis was performed as recommended by the Cochrane handbook in the following manner: tOC and ucOC within the comparison of GDM and nondiabetic pregnant controls. The effect size was expressed as mean difference (MD) by random effects models (REM) weighting by the inverse of the variance since all studies used randomly sampled [41].

Results are presented using forest plots including the MDs for the main groups (cases and controls). For subgroup analysis, a mixed effects model (MEM) was employed [42].

Between-study variability was assessed using the *τ* ^2^ and Cochran's Q and *I* ^2^ statistics [43]. The contribution of individual-study heterogeneity was visually assessed by Baujat plots [44].

Multiple metaregressions were performed to add another approach for unexplained heterogeneity and to determine which variables influenced tOC and ucOC serum levels. When enough information was available, we used the following covariates: mean gestational age at inclusion; mean maternal age; body mass index; mean weight gain at inclusion; mean serum levels of triglycerides; mean serum levels of fasting glucose; mean serum levels of fasting insulin; mean latitude; mean altitude; and UV where the study was conducted. The UV index was classified as follows: 1–2 low, 3–5 moderate, 6–7 high, 8–10 very high, and ≥11 extremely high. *I* ^2^ and *R* ^2^ values were reported to present residual heterogeneity and the amount of heterogeneity explained by each variable, respectively.

Publication bias was visually assessed by contour-enhanced funnel plots and quantified by Egger method. Also, in order to assess “small-study effect” defined as the chance of finding a trend towards a larger effect due to the higher probability of a small study of being published when a more “significant” result is found, a cumulative analysis was performed and presented as a forest plot [45, 46]. A sensitivity analysis was performed to assess the pooled estimates according to study quality. Statistical analysis was conducted using R studio v1.0.136 (The R Foundation for Statistical Computing) [package “meta v4.2”] [47].

## 3. Results

### 3.1. Study Selection and Study Characteristics

**Figure 1:**
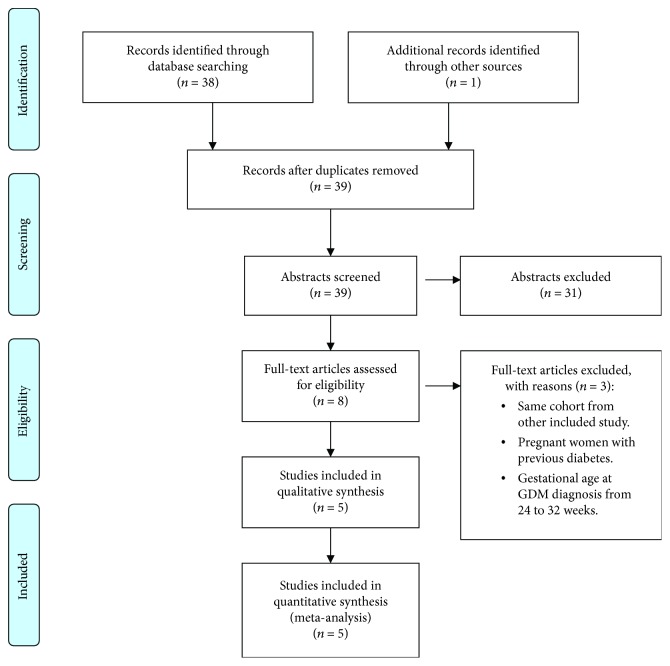
PRISMA flow diagram of included studies.

A total of 38 studies were identified by database searching, with one additional study included manually. Of them, eight studies were eligible for full-text review. After review, five studies [32, 33, 48–50] were retained for the systematic review and meta-analysis.  Figure 1 depicts the review flow diagram.

Reasons for excluding three full-text studies were as follows: one of them used the same cohort of another included study in our analysis [51], while the second one performed the diagnostic tests for GDM from 24 to 32 weeks of gestation [52]. The last excluded study was due to inclusion of women with a diagnosis of diabetes before pregnancy [53]. The following authors were reached by mail and they provided aggregated data on their published studies: Tabatabaei et al. [49], Saucedo et al. [33], and Srichomkwun et al. [50].  Annex 2 in the supplemental material details the shared information. Also, the characteristics of the included articles are described in Table 1.

We used the Newcastle-Ottawa scale for study-quality assessment in observational studies. From a total of 9 possible rating points, three studies [32, 48, 50] presented 6 points mainly due to lack of representativeness of the cases, lack of nonresponse rate description, or no description for control selection. Two studies [33, 49] had 7 points or more due to the representativeness of the cases, study controls for each additional outcome, or lack of nonresponse rate description. Tables 2 and 3 show the full Newcastle-Ottawa scale assessment. From the five included studies [32, 33, 48–50], a total of 1119 pregnant women underwent assessment for OC serum concentrations; from these, 23% (259/1119) were diagnosed as GDM. The mean reported maternal age among studies was 30.5 (SD 2.3) years. Also, the mean BMI was 27.3 (SD 3) kg/m^2^ with a weight gain of 11.7 (SD 3.7) kg. Mean gestational age at diagnosis of GDM was 26.6 (SD 1.5) weeks; this was also the gestational age at enrollment and measurement of OC.

### 3.2. Total Osteocalcin among Gestational Diabetes Mellitus

**Figure 2:**
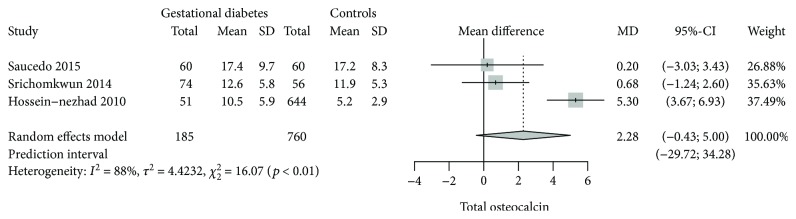
Forest plot on the mean difference of tOC among GDM and nondiabetic pregnant controls.

Three studies [32, 33, 50] had information regarding tOC concentrations among GDM. There was no statistically significant mean difference between women with GDM and nondiabetic pregnant controls (2.28; 95% CI: −0.43 to 5.00; *p* = 0.099). A *Q* value of 4.42 with 2 degrees of freedom and *p* < 0.01 provides evidence that the effect size varies across studies. *I* ^2^ indicates that 88% of the variation can be attributed to true effect rather than random error (Figure 2).

**Figure 3:**
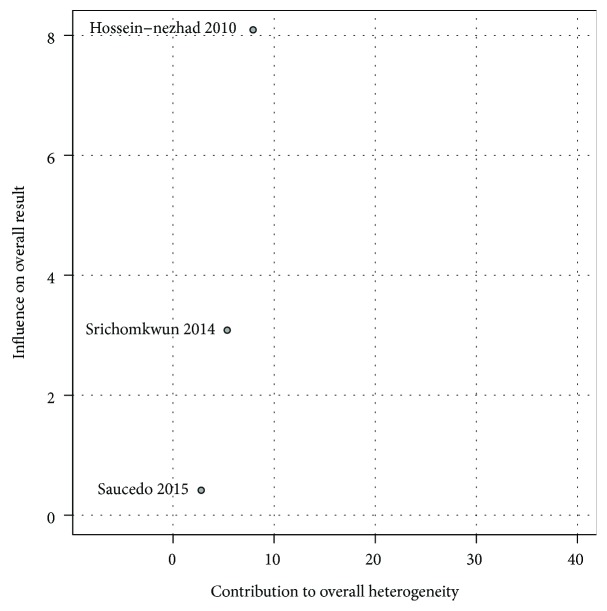
Baujat plot for the mean difference and contribution to heterogeneity among studies measuring tOC.

Baujat plot showed that the study from Hossein-nezhad et al. [32] contributed the most to the overall heterogeneity and influenced the most to the overall results (Figure 3).

**Figure 4:**
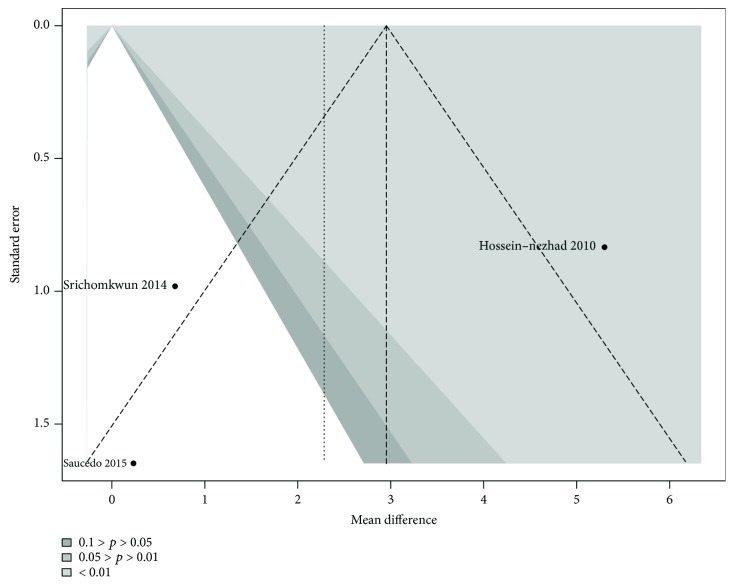
Funnel plot for publication bias assessment among studies measuring tOC.

Contour-enhanced funnel plot showed no significant publication bias (Figure 4), also quantified by Egger method (estimate: −0.670; *p* = 0.119).

**Figure 5:**
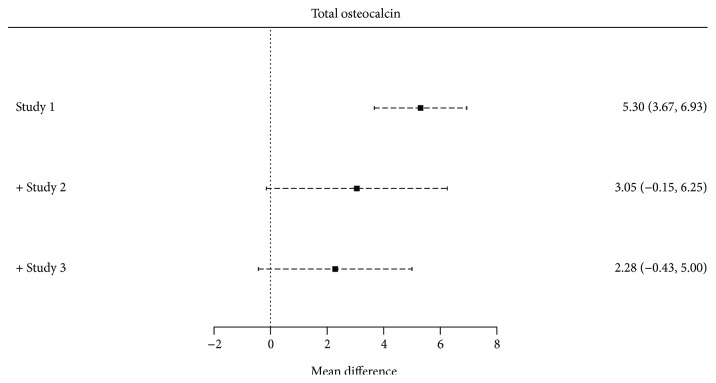
Cumulative forest plot among studies measuring tOC.

According to the cumulative analysis, there was no trend towards more significant results in smaller studies, reducing the probability of publication bias (Figure 5).

### 3.3. Subgroup Analysis for tOC

Formal assessment of heterogeneity could not be performed by subgroup analysis since all studies differed among solar exposition, brand, and method for OC measurement.

### 3.4. Metaregressions for tOC

**Figure 6:**
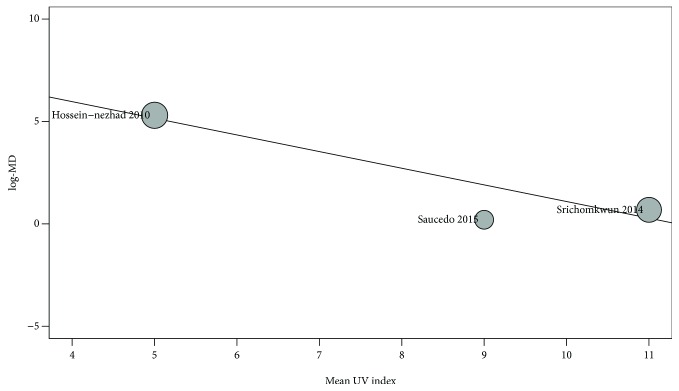
Metaregression on the pooled mean difference for tOC and the mean UV index.

Several covariates were used to assess their influence on the mean serum concentrations of OC. Mean UV index was the only variable significantly influencing serum levels of OC; the higher the UV index, the lower the mean difference of tOC between women with GDM and pregnant controls (estimate: −0.812; 95% CI: −1.22 to −0.39; *p* < 0.001). The effect of UV index accounted for 100% of the heterogeneity in this sample (*R* ^2^ = 1.0), leaving no residual heterogeneity (*I* ^2^ = 0%).  Figure 6 shows the metaregression for mean UV index.

### 3.5. Undercarboxylated Osteocalcin among Gestational Diabetes Mellitus

**Figure 7:**
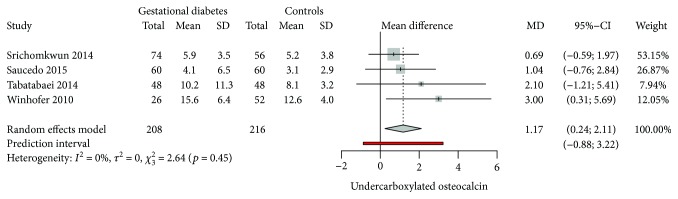
Forest plot on the mean difference of ucOC among GDM and nondiabetic pregnant controls.

Four studies [33, 48–50] had information regarding ucOC concentrations among GDM. Mean serum levels of ucOC were significantly higher in women with GDM when compared to nondiabetic pregnant women (1.17; 95% CI: 0.24 to 2.11; *p* = 0.013). *Q* value of 2.64 with 3 degrees of freedom and *p* = 0.45 provides evidence that the true-effect size does not significantly vary across studies. *I* ^2^ also depicts this true-effect variability to be 0% (Figure 7).

**Figure 8:**
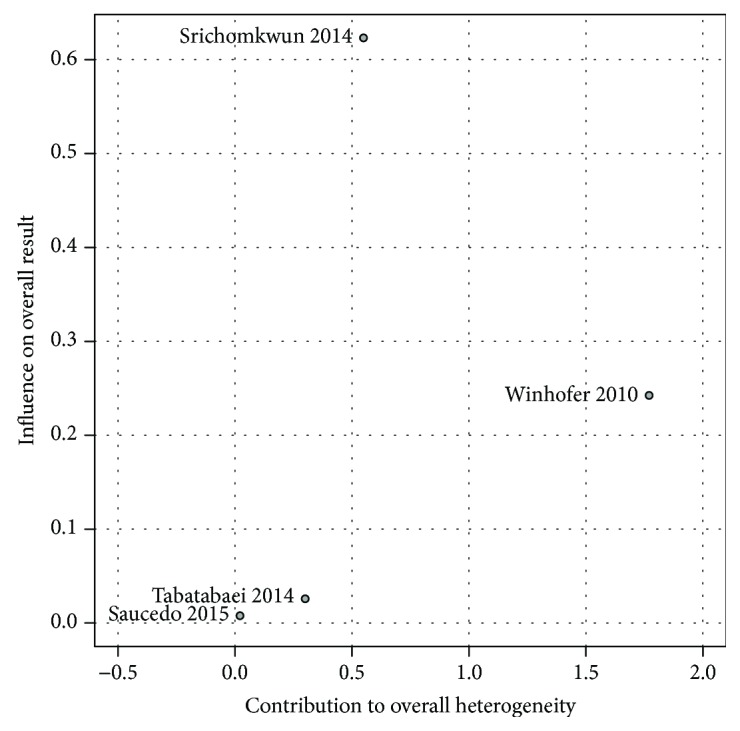
Baujat plot for the mean difference and contribution to heterogeneity among studies measuring ucOC.

Prediction interval from −0.88 to 3.22 shows the probability of a future ucOC measurements to lie within this range. Baujat plot showed that the study from Winhofer et al. [48] contributed the most to the overall heterogeneity among studies, while the study from Srichomkwu et al. [50] influenced the most to the overall results (Figure 8).

**Figure 9:**
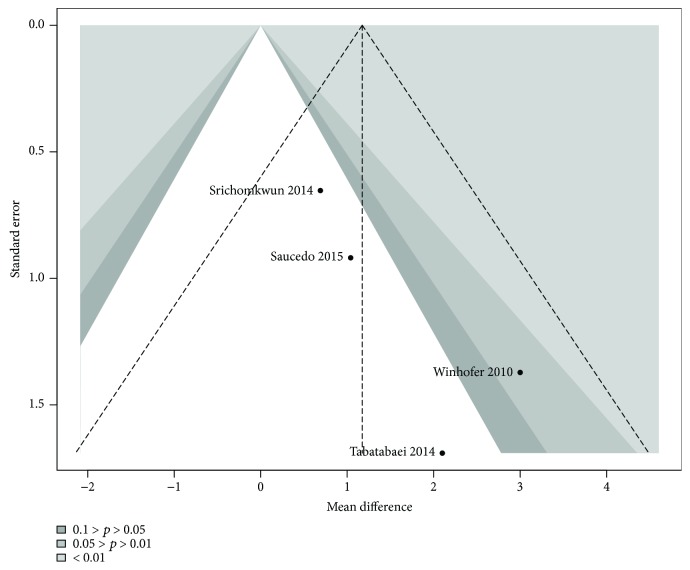
Funnel plot for publication bias assessment among studies measuring ucOC.

Contour-enhanced funnel plot showed no significant publication bias (Figure 9), also quantified by Egger method (estimate: −0.670; *p* = 0.119).

**Figure 10:**
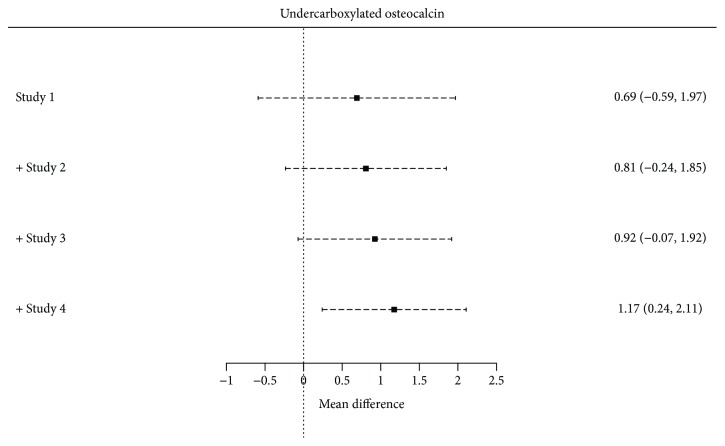
Cumulative forest plot among studies measuring ucOC.

According to the cumulative analysis, there was a trend towards more significant results in smaller studies, increasing the probability of publication bias (Figure 10).

### 3.6. Subgroup Analysis for ucOC

**Figure 11:**
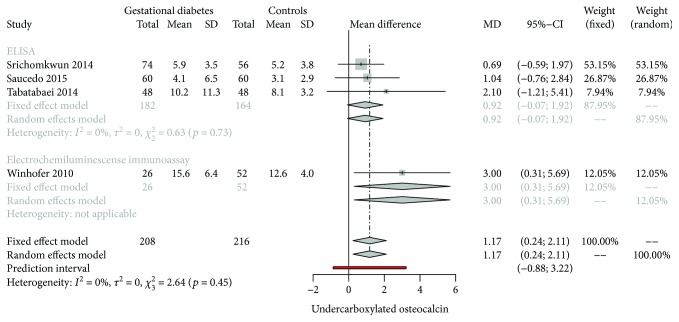
Forest plot for subgroup analysis on the methods used for measuring ucOC.

Formal assessment of heterogeneity showed that the use of ELISA as the method for ucOC analysis depicted similar results between studies. No added variability was found in this subgroup (*I* ^2^ = 0%).  Figure 11 shows the forest plot for method analysis. Also, no covariate significantly influenced the pooled mean difference for ucOC.

## 4. Discussion

### 4.1. Main Findings

There are some crucial points elucidated by the present study. (1) There is no significant difference in tOC concentrations among women with GDM and nondiabetic pregnant controls during the second trimester (at time of GDM screening). (2) Serum concentrations of ucOC are significantly higher in women with GDM when compared to nondiabetic controls. (3) The only extrinsic factor influencing the serum concentrations of tOC is the UV index, showing an inverse correlation between the mean difference in tOC and the level of exposition to UV rays. (4) There was no intrinsic factor such as maternal age, BMI, fasting insulin, fasting glucose, triglycerides, or gain weight during pregnancy, significantly influencing the mean difference of ucOC or tOC. Finally, (5) ELISA as a method for measuring ucOC was the only one comparable among studies, showing no heterogeneity and yielding similar results.

The molecular mechanisms underlying OC differences among pregnant and nonpregnant women have not been yet described. One of the reasons why OC may be higher among women with GDM is the fact that placental-induced insulin resistance reaches its peak between 24 and 28 weeks of gestation. This insulin resistance leads to an increase in insulin secretion by pancreatic B-cells as a compensatory mechanism that derives in an increased anabolic feature on bone metabolism via IGF-1, therefore, influencing OC concentrations especially during the second trimester of pregnancy as described by Winhofer et al. [48].

Similar to our results, the study of Telejko et al. [52] showed no significant difference in tOC levels among women with and without GDM. This study was excluded due to the inclusion of women after 28 weeks of gestation as defined previously. The study conducted by Martinez et al. [53] showed an increase of tOC concentrations among women with diabetes, which was also discarded from our meta-analysis due to the inclusion of prediabetic patients.

### 4.2. Clinical Implications

Because GDM is usually diagnosed late in pregnancy, early preventive strategies are precluded. Indeed, there is good evidence that timely diagnosis and treatment of GDM by dietary advice, blood glucose monitoring, and insulin therapy when needed significantly reduce perinatal complications such as preeclampsia or macrosomia [54, 55]. Furthermore, there is mounting evidence that lifestyle interventions early in pregnancy in high-risk women reduce the risk of developing GDM and its associated complications [56, 57]. Thus, developing predictive models for GDM is a challenge in contemporary fetal-maternal medicine. However, the models described so far have limited predictive capacity. At least five predictive models are aiming to assess the predictive performance for GDM during the first trimester based on maternal characteristics, but none of them have found more than 55% prediction based on these variables. Also, their described AUC ranged from 0.703 to 0.832 [58–62], showing modest overall results. Our study suggests that OC levels may have the potential to improve current predictive strategies based on previous maternal characteristic models.

### 4.3. Strengths and Limitations

There are two main strengths of our study: firstly, we conducted a rigorous systematic review by independent reviewers and a third one for evaluation, assessment of bias, and database searching was also done by independent investigators all blinded to authorship and hospital where the study was conducted, allowing us to minimize bias when selecting publications for inclusion. Finally, an exhaustive analysis for assessment of intrinsic and extrinsic factors was performed to determine the influence that each one had on the mean difference between GDM and controls.

The main limitation of this study is the small number of publications found in the literature. This limitation is not due to the search strategy but to the real limited number of published papers aiming to measure OC in GDM. Also, there are other causes that may influence OC concentration among women with gestational diabetes such as thyroid diseases or smoking status, but due to the limited information of all possible causes described in each study, it was implausible for us to analyze other causes on individual basis.

### 4.4. Osteocalcin as a Predictive Protein for Gestational Diabetes Mellitus

As mentioned before, OC is implicated not only in bone metabolism but in the function of the whole body by exerting its effect when binding to the GPRC6A receptor [28, 63, 64]. The potential role for OC as a predictive protein for GDM comes from previous studies relating knockout mice for GPRAC6A (OC receptor) and later smaller pancreatic islet size, lower insulin content, lower pancreatic weight, lower number of islets, lower insulin mRNA expression, and lower insulin secretion in response to osteocalcin with glucose intolerance [65]. Also, the study of Papastefanou and colleagues [31] reported that an elevation of tOC serum levels in maternal serum during the first trimester of gestation is a significant predictor for GDM as a standalone parameter (AUC 0.61) or in combination with maternal and pregnancy characteristics (AUC 0.80). Our study adds to the current knowledge by showing that ucOC is the type of OC that has more pronounced changes in women with GDM. The next research step would be to assess if first-trimester ucOC adds to other existing algorithms in predicting GDM [35].

## 5. Conclusions and Implications

This meta-analysis provides evidence to support the use of ucOC as a potential marker for GDM rather than tOC, probably because the latter is more influenced by baseline UV exposure. In addition, our study also suggests that measuring ucOC during pregnancy also has the advantage of reduced variability, regardless of the platforms or methods used.

## Figures and Tables

**Table 1:** Characteristics of included studies.

Study	Year	Country	Type of study	Inclusion criteria	Exclusion criteria	Diagnostic criteria for GDM	Type of osteocalcin	Analysis method	Study participants	Number of nondiabetic women	Number of diabetic women
Winhofer	2010	Iran	Case control	Healthy pregnant women, who were referred from the department of obstetrics and gynecology to the outpatient clinic for routine glucose tolerance testing between the 24th and 28th gestational weeks	History of GDM or obstetric complications in a previous pregnancy, any history of impaired glucose tolerance, or signs of fetal macrosomia. Previous OGTT during pregnancy	One-step: 75 g of glucose (5.5, 10.0, and 7.8 mmol/l cutoff at 0, 1, and 2 h)	ucOC	ELISA for tOC	78	52	26

Hossein-nezhad	2010	Iran	Case control	Pregnant women without previous history of diabetes mellitus who referred to the clinics for prenatal care during the first half of the pregnancy	None	Two-step: 50 g if >7.2 mmol/L, 100 g	tOC	ELISA	695	644	51

Tabatabaei	2014	Canada	Cohort	Healthy pregnant Caucasian women and women with GDM having singleton and term pregnancies with gestation duration of 37 to 42 weeks. With high risk of GDM (obesity and previous history of GDM)	History or new diagnosis of corticosteroid-treated asthma, liver disease, renal disease, Crohn's disease, ulcerative colitis, celiac disease, medication known to affect bone metabolism, and history of type 1 diabetes or type 2 diabetes	Two-step: 50 g if >7.2 mmol/L, 100 g	ucOC	ELISA	96	48	48

Saucedo	2015	Mexico	Case control	Nongestational diabetic women and gestational diabetic women screened at 24–28 weeks	History of arterial hypertension, renal disease, liver disease, thyroid disorders, and other endocrine or chronic diseases	Two-step: 50 g if >7.2 mmol/L, 100 g	tOC & ucOC	IRA for tOC & ELISA for ucOC	120	60	60

Srichomkwun	2015	Thailand	Case control	Pregnant women without risk of gestational diabetes mellitus	History of DM before pregnancy, twin or multiple pregnancy, or significant medical problem, such as cardiovascular, liver, or renal disease	Two-step: 50 g if >7.2 mmol/L, 100 g (cutoff Carpenter Coustan 5.3, 10.0, 9.2, and 8.1 mmol/L at 0, 1, 2, and 3 h	tOC & ucOC	ELISA	130	56	74

tOC: total osteocalcin; ucOC: undercarboxylated osteocalcin; ELISA: electrochemiluminescence immunoassay; IRA: immunoradiometric assay; GDM: gestational diabetes mellitus.

**Table 2:** Newcastle-Ottawa scale for case-control studies.

Study		Selection			Definition of controls	Comparability		Exposure			Stars
Author	Year	Is the case definition adequate?	Representativeness of the cases	Selection of controls		Study controls for main outcome	Study controls for additional outcomes	Ascertainment of exposure	Same method of ascertainment for cases and controls	Nonresponse rate	
Winhofer	2010	∗		∗	∗	∗		∗	∗		6
Saucedo	2015	∗		∗	∗	∗		∗	∗	∗	7
Tabatabaei	2014	∗	∗	∗	∗	∗		∗	∗		7
Srichomkwun	2015	∗	∗			∗		∗	∗	∗	6

**Table 3:** Newcastle-Ottawa scale for cohort studies.

Study		Selection			Demonstration that outcome of interest was not present at start of study	Comparability		Exposure			Stars
Author	Year	Representativeness of the exposed cohort	Selection of the nonexposed cohort	Ascertainment of exposure		Study controls for main outcome	Study controls for additional outcomes	Ascertainment of outcome	Was follow-up enough for outcomes to occur	Adequacy of follow-up cohorts	
Hossein-nezhad	2010	∗	∗	∗	∗	∗		∗			6

## Additional Points

*Condensation*. This meta-analysis provides evidence to support the use of ucOC as a potential marker for GDM rather than tOC, yielding very little variability among studies and no difference among methods or brands used for its analysis.

## Supplementary Materials

Supplementary 1 Annex 1: query syntax for data search.

Supplementary 2 Annex 2: shared information from other authors.
