# Supplementary material for: Osteocalcin Serum Levels in Gestational Diabetes Mellitus and Their Intrinsic and Extrinsic Determinants: Systematic Review and Meta-Analysis
Source: J Diabetes Res. 2018 Dec 30;2018:4986735. doi: 10.1155/2018/4986735 (PMC6332945; doi:10.1155/2018/4986735)
Supplement: Supplementary 1 — Annex 1: query syntax for data search. [file 4986735.f1.docx]

Query:

osteocalcin gestational diabetes= 14 results

("osteocalcin"[MeSH Terms] OR "osteocalcin"[All Fields]) AND ("diabetes, gestational"[MeSH Terms] OR ("diabetes"[All Fields] AND "gestational"[All Fields]) OR "gestational diabetes"[All Fields] OR ("gestational"[All Fields] AND "diabetes"[All Fields]))

osteocalcin pregnancy diabetes

(("osteocalcin"[MeSH Terms] OR "osteocalcin"[All Fields]) AND ("pregnancy in diabetics"[MeSH Terms] OR ("pregnancy"[All Fields] AND "diabetics"[All Fields]) OR "pregnancy in diabetics"[All Fields] OR ("pregnancy"[All Fields] AND "diabetes"[All Fields]) OR "pregnancy diabetes"[All Fields])) AND "humans"[MeSH Terms]

Ebsco=10 results

osteocalcin AND gestational diabetes mellitus

Language: english

Query

IWS

14 results

osteocalcin gestational diabetes mellitus)

*Index=SCI-EXPANDED, SSCI, A&HCI, BKCI-S, BKCI-SSH, ESCI Time period=All years*
