# Supplementary material for: Osteocalcin Serum Levels in Gestational Diabetes Mellitus and Their Intrinsic and Extrinsic Determinants: Systematic Review and Meta-Analysis
Source: J Diabetes Res. 2018 Dec 30;2018:4986735. doi: 10.1155/2018/4986735 (PMC6332945; doi:10.1155/2018/4986735)
Supplement: Supplementary 2 — Annex 2: shared information from other authors. [file 4986735.f2.docx]

Saucedo, R., Rico, G., Vega, G., Basurto, L., Cordova, L., Galvan, R., . . . Zarate, A. (2015). Osteocalcin, under-carboxylated osteocalcin and osteopontin are not associated with gestational diabetes mellitus but are inversely associated with leptin in non-diabetic women. *J Endocrinol Invest, 38*(5), 519-526. doi:10.1007/s40618-014-0220-4

|  | GDM | Controls |
| --- | --- | --- |
| tOC (ng/mL) | 17.4 ± 9.7 | 17.2 ± 8.3 |
| ucOC (ng/mL) | 4.14 ± 6.5 | 3.1 ± 2.9 |

Tabatabaei, N., Giguere, Y., Forest, J. C., Rodd, C. J., Kremer, R., & Weiler, H. A. (2014). Osteocalcin is higher across pregnancy in Caucasian women with gestational diabetes mellitus. *Can J Diabetes, 38*(5), 307-313. doi:10.1016/j.jcjd.2014.02.007

|  | GDM | Controls |
| --- | --- | --- |
| ucOC (nmol/l) | 1.74 ± 0.31 | 1.38 ± 0.11 |

Srichomkwun, P., Houngngam, N., Pasatrat, S., Tharavanij, T., Wattanachanya, L., & Khovidhunkit, W. (2016). Undercarboxylated osteocalcin is associated with insulin resistance, but not adiponectin, during pregnancy. *Endocrine, 53*(1), 129-135. doi:10.1007/s12020-015-0829-x

|  | GDM | Controls |
| --- | --- | --- |
| tOC (ng/mL) | 12.6 ± 5.82 | 11.92 ± 5.43 |
| ucOC (ng/mL) | 5.89 ± 3.55 | 5.20 ± 3.79 |
